# Supplementary material for: Neural-like computing with populations of superparamagnetic basis functions
Source: Nat Commun. 2018 Apr 18;9:1533. doi: 10.1038/s41467-018-03963-w (PMC5906599; doi:10.1038/s41467-018-03963-w)
Supplement: Supplementary file 1 — Supplementary Information [file 41467_2018_3963_MOESM1_ESM.pdf]

SUPPLEMENTARY INFORMATION

Supplementary Note 1: using spin-orbit torques to shift the junctions

In order to induce shifts through spin-orbit torques, the junctions should be grown from the free layer to the pinned layer, on top of a heavy metal underlayer with variable width, as shown in Supplementary Fig. 1. When a current I_{SOT} is injected in the underlayer, spin-orbit torques influence the magnetization of the free layer and modify the spin transfer term in the expression of the switching rates[1]. This is equivalent to biasing the tuning curve with a voltage proportional to the current density in the metallic layer. As the width of the metallic layer is different for each junction, the effective bias is different. The frequency of a junction located above an underlayer of width w is:

$$F(V,w) = \frac{1}{\tau_0 \exp\left(\frac{\Delta E}{k_B T}\right) \cosh\left(\frac{\Delta E}{k_B T}\left(\frac{V_{STT}}{V_c} + \frac{d\, t_j I_{SOT}}{w\, t_u\, I_c}\right)\right)}, \quad (1)$$

In this expression, V_{STT} is the voltage stimulus, applied through a common voltage to all the junctions. I_c is the critical current linked to spin transfer torque, d is the diameter of the junction, t_j is the thickness of its free layer and V_c is the critical voltage linked to spin orbit torque. Through spin orbit torque, the injected current in the underlayer I_{SOT} induces a shift of the tuning curve *F(V,w)*, which depends on the width w of the heavy metal underlayer and its thickness t_u. Choosing carefully the shape of the heavy metal underlayer can then allow shifting differently the different junctions located on top and building a population of junctions all tuned to different voltages.

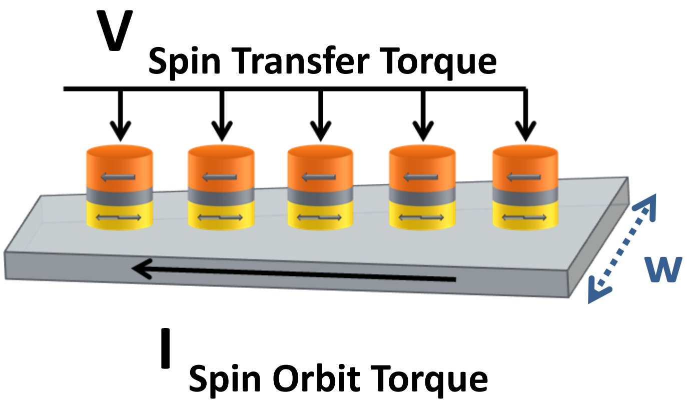

Supplementary Figure 1: Schematic of a hardware implementation of a population using spin-orbit torque.

Supplementary Note 2: robustness to variability

2.1 Robustness to variability in the junctions critical voltage

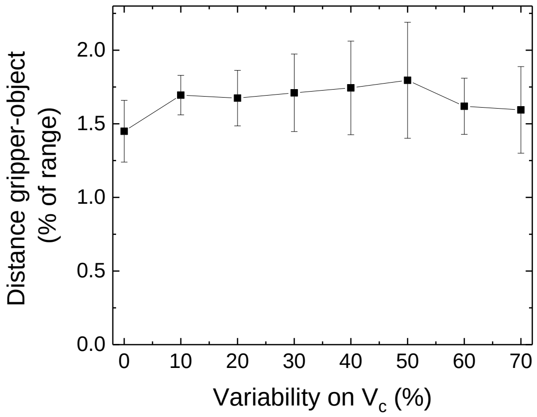

Supplementary Figure 2: Distance between the gripper and the object as a function of variability in the junctions critical voltage. Each population is composed of 100 junctions and 3,000 learning steps were used. Each data point is the average over 5 trials and the error bar is the corresponding standard deviation.

Supplementary Fig. 2 shows the effect of variability in the junctions critical voltage on the precision of the system. We observe a strong robustness to this variability. Indeed, the width of the tuning curves is proportional to V_c, and in consequence random variations of V_c do not affect the average width of the tuning curves and thus the precision of the coding.

2.2 Robustness to variability in the energy barrier

As can be seen from Fig. 3e of the main paper, not only is our system robust to variability; it is improved by a small amount of variability. This can be interpreted as follows. The variability on the energy barrier has a uniform distribution between $\Delta E_0 - \sigma$ and$\Delta E_0 + \sigma$. The average frequency of a junction is:

$$< F > = < \frac{1}{2\tau_0} \exp\left(-\frac{\Delta E}{k_B T}\right) > = \frac{1}{2\tau_0} \exp\left(-\frac{\Delta E_0}{k_B T}\right) \frac{\sinh \sigma}{\sigma} = F_0 \frac{\sinh \sigma}{\sigma}. \quad (2)$$

Therefore, the average frequency $< F >$ is higher than the theoretical frequency F_0 and the precision is increased. When the variability is too high, the mismatch between the expected theoretical tuning curves and the observed tuning curve is too important so the precision is worse than without variability.

Supplementary Note 3: resilience to the loss of neurons

In this section, we investigate the effect on our system of the loss of neurons. In the case of superparamagnetic tunnel junctions, this loss corresponds to a breakdown of the tunnel barrier: the resistance of the junction drops and the oscillations of the free magnetic layers are not detected anymore, leading to an effective zero rate.

We consider the case of a system with an input population and an output population, each constituted of 100 junctions such that $\frac{\Delta E}{k_{\mathrm{B}}T} = 6$ and the critical voltage $V_{\mathrm{c}} is\ 0.1\ \mathrm{V}$. The system is trained as described in the main text, with 3000 learning steps. Then, a certain percentage of the neurons are killed by setting the rates of randomly chosen junctions to zero.

The open circles in Supplementary Fig. 3 presents the evolution of the distance gripper-target versus the number of re-learning steps after the loss, for various levels of loss (various colors). As can be expected, the distance gripper-target increases with the number of lost neurons. We observe that even without re-learning after the loss, the distance gripper-target is much smaller than in the case of an untrained network (see Fig. 3a in the main text). This highlights the resilience of population coding to the loss of neurons. We observe that re-learning allows decreasing significantly the distance gripper-target after the loss: the system recovers.

We compare this re-learning with initial learning. To do this we apply the loss of neurons to the system before training it, and then execute the learning. The distance gripper-target is plotted versus the number of learning steps with full squares in Supplementary Fig. 3. The various colors correspond to the levels of loss and match with the post-learning loss configuration. We observe that the final distance gripper-target obtained by initial learning and by re-learning match, for each level of loss.

However, the number of steps required for the system to recover is significantly smaller than the number of steps required for initial learning (several hundred steps versus several thousand). This highlights how our computing can quickly adapt to drastic changes.

To conclude, these results demonstrate the resilience of our system to faulty superparamagnetic tunnel junctions.

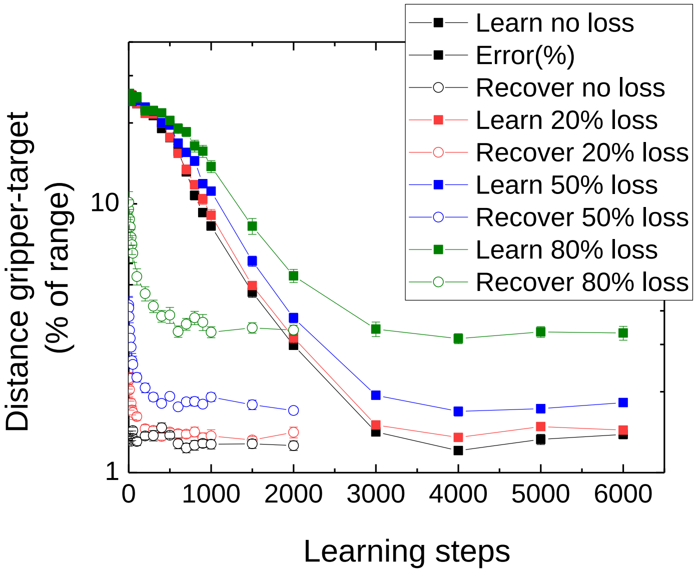

Supplementary Figure 3: Distance gripper-target versus the number of learning steps for various configurations.

The open circles correspond to the configuration where the system has been trained, then submitted to loss, and represent the re-learning. The full squares correspond to the configuration where the system was submitted to loss, then trained. The various colors correspond to various levels of loss. Each data point corresponds to an average over 50 trials and the error bar (in most cases smaller than the marker) to the single standard deviation in the mean.

Supplementary Note 4: schematic of systems with two inputs and cascaded non-linear operations

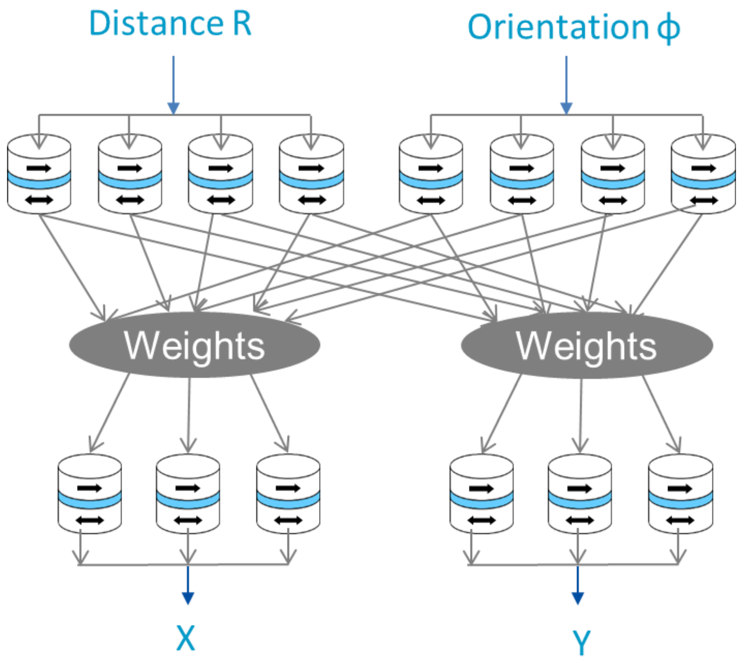

Supplementary Figure 4: Schematic of the two-inputs system allowing for the transformation from polar to Cartesian coordinates. Each output population X and Y is linked to both input populations R and ϕ. This corresponds to the label 2-Inputs in Fig. 4a of the main text.

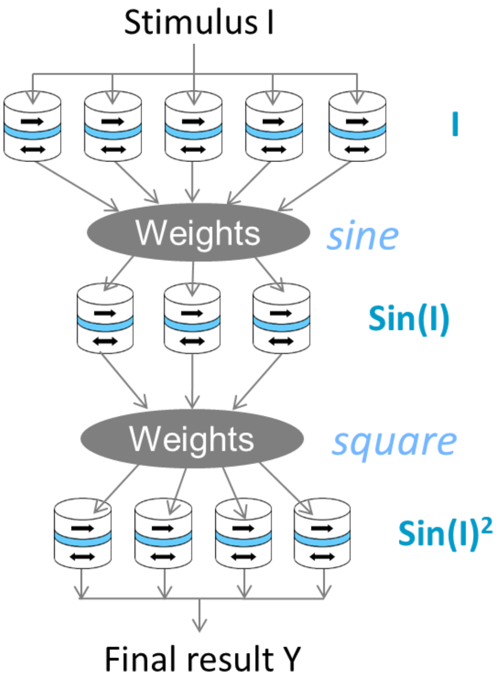

Supplementary Figure 5: Schematic of the system allowing for the composed function (Sine)^2. A first set of weights produces the transformation sine, then a second set of weight produces the transformation square. This corresponds to the label Series in Fig. 4a of the main text.

Supplementary Note 5: data path of the full system

Simplified diagram

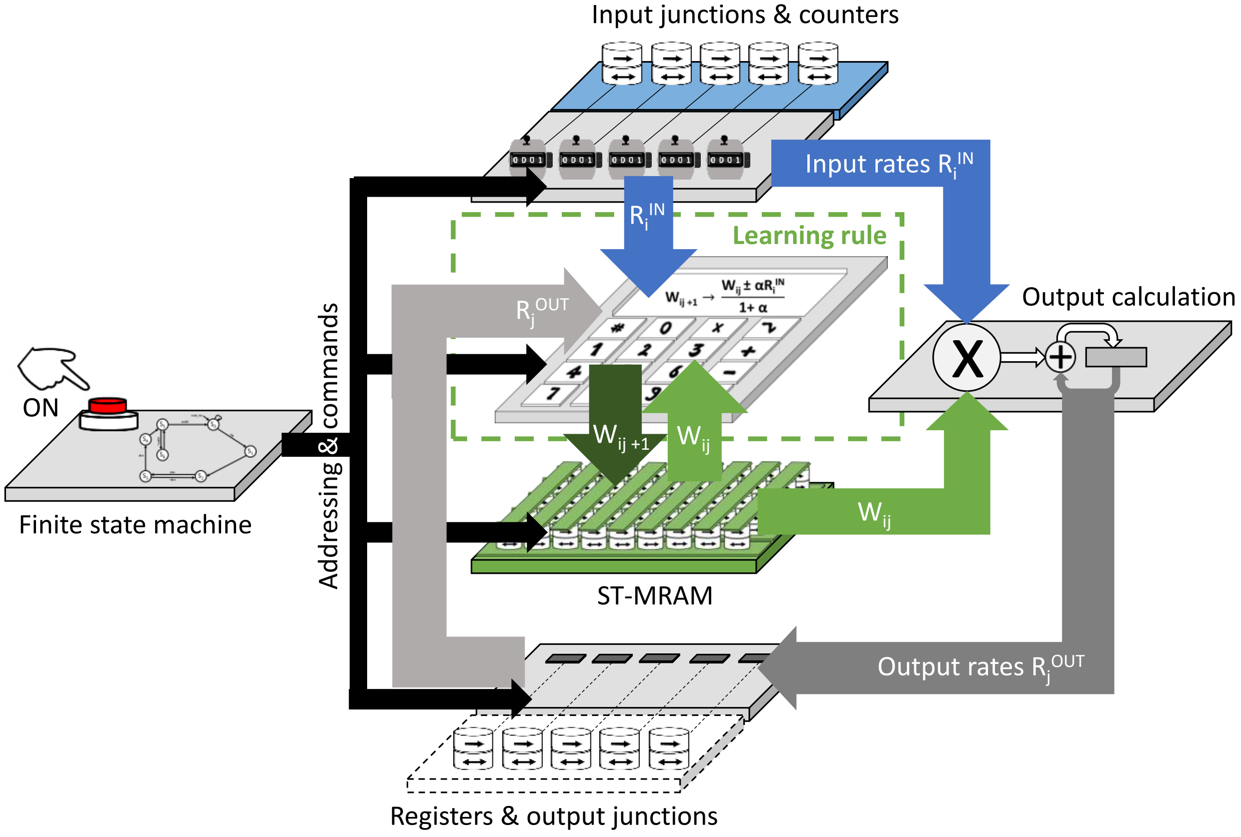

Full datapath

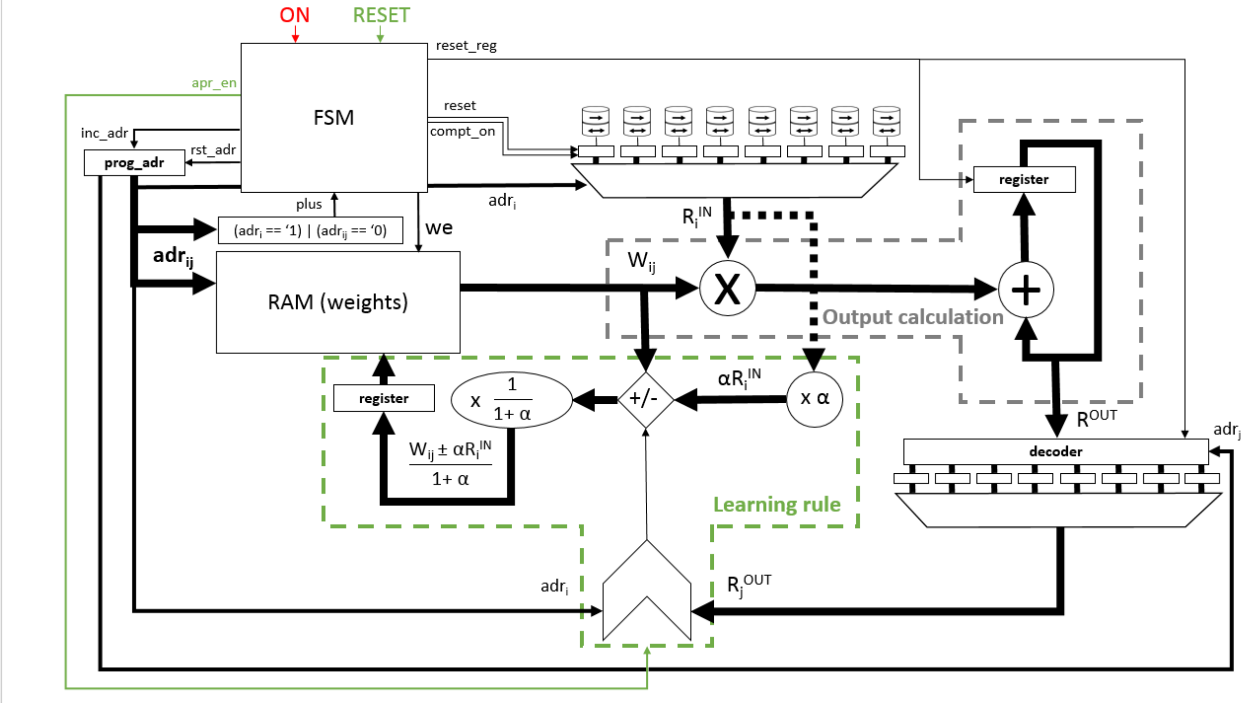

Supplementary Figure 6: (a) Schematic of the data path of the full system (in simplified and more comprehensive forms), associating superparamagnetic junctions, weights implemented using ST-MRAM, and CMOS circuitry.
Meaning of the abbreviations. RAM: random access memory. FSM: finite state machine. *we: write enable, adr: addres*. Arithmetic operations are realized in Fixed Point representation (integer arithmetics).

Before the operation of the system, weights in ST-MRAM memory are programmed with random values.

The general principles of this system are described in the Methods section of the article.

The operating steps of the finite state machine (FSM) are as follows:

1. As long as the state machine is in state S0, each counter at the output of each superparamagnetic tunnel junction computes the number of junction switches.
2. When the ON signal is enable, the counting phase stops and the computation of the r^{out} values with equation (4) (from the main article) starts. This computation involves three states S1, S2 and S3 of the finite state machine. The values of the input counters R_i^{IN} are multiplied with weights *Wij* stored in RAM sequentially and added to obtain the result of equation (4). Address increment is performed automatically, and the resulting R_{out} values are stored in registers.
3. If the system is in a learning phase, a consecutive learning phase allows updating the value of the weights in the ST-MRAM following the learning rule described in the main article. The outputs R_j^{OUT} are compared with input addresses, therefore controlling the updating process of the weights. The computation is done by addition and multiplication involving the different parameters of the system. All these operations involve three states S4, S5 and S6 of the state machine.

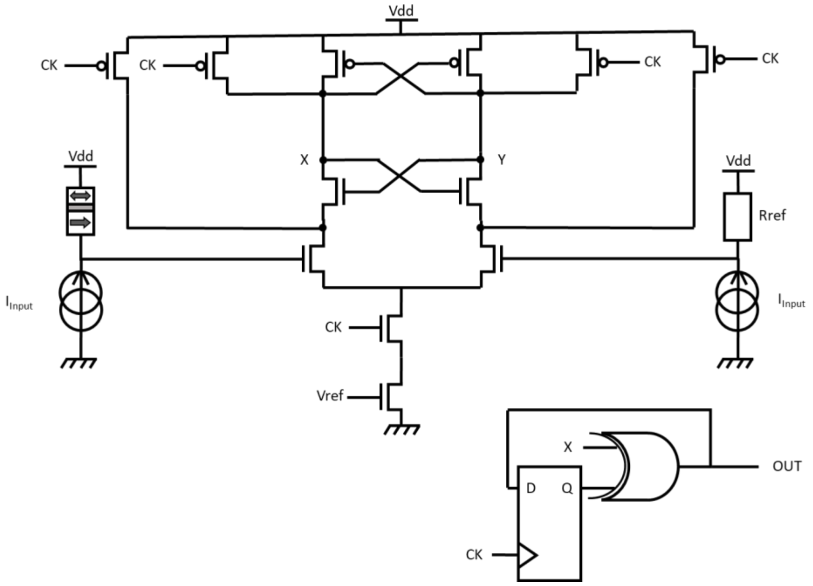

Supplementary Figure 7: Circuit for converting the switching events of a superparamagnetic tunnel junction to a CMOS digital signal.
In addition to the superparamagnetic tunnel junction, the stimulus current is applied to a reference resistor R_{ref}, whose resistance is intermediate between the parallel and anti-parallel state resistance of

the superparamagnetic tunnel junctions, and at each clock cycle, the voltage at the junction and at the reference resistor is compared by a low power CMOS comparator. Simple logic comparing the result of the comparison to the same result at the previous clock cycle allows detecting the junction switching events, which are counted by an eight-bit digital counter.
This design is not able to detect multiple switching occurring during a single clock cycle. We saw on system-level simulations that this particularity has no impact on the full application.

Supplementary Note 6: area and energy efficiency of variations of the full system

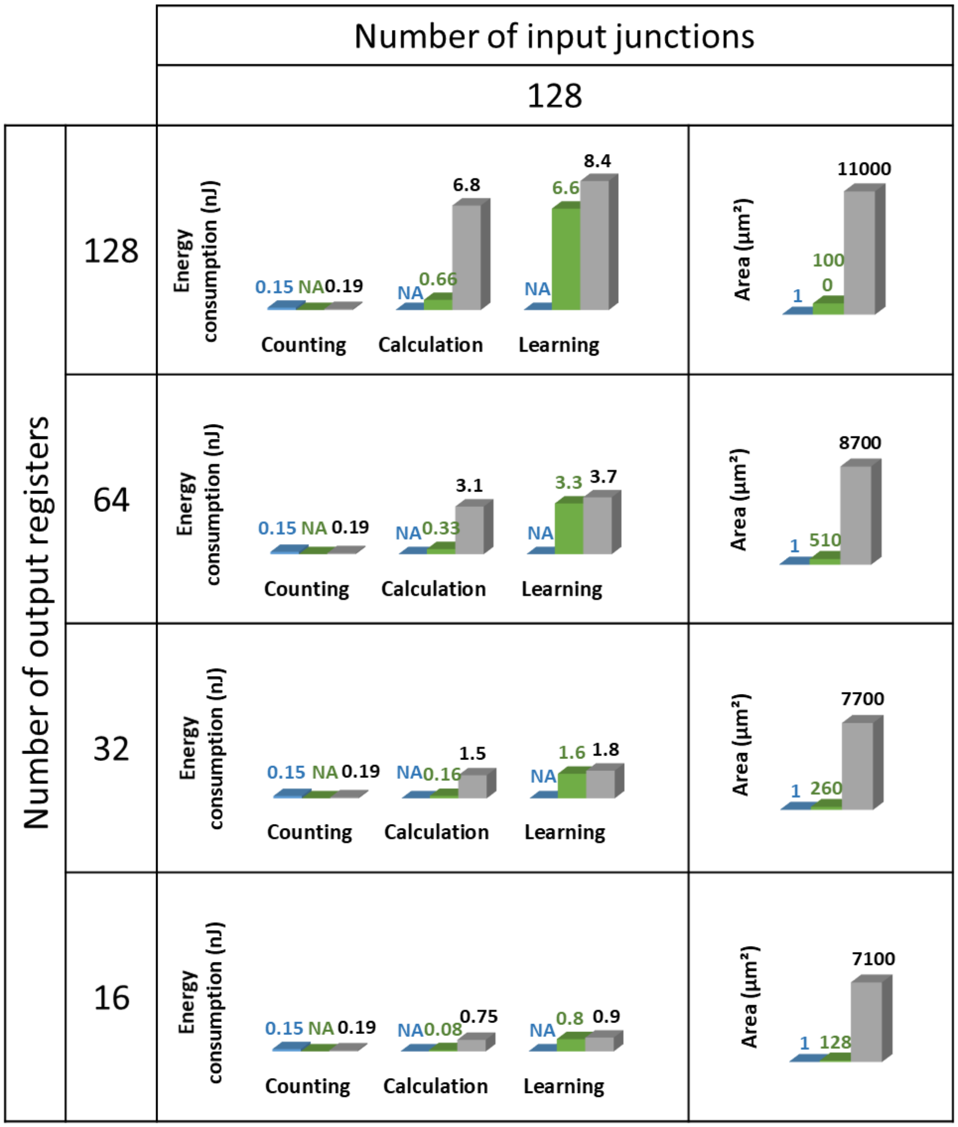

Supplementary Figure 8: Energy consumption and area occupied by systems with 128 input junctions, and 128, 64, 32 or 16 outputs.
Color code (shared with Fig. 5 in the main article). Blue : superparamagnetic junction. Green : ST-MRAM. Grey : CMOS circuits.

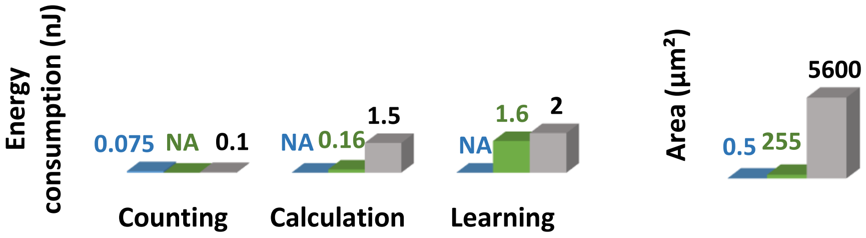

Supplementary Figure 9: Energy consumption and area occupied by a system with 64 input junctions, and 64 outputs.

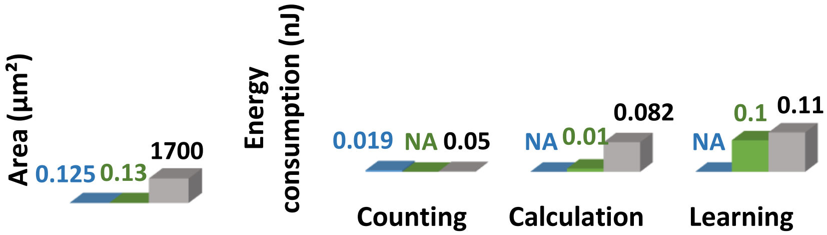

Supplementary Figure 10: Energy consumption and area occupied by a system with 16 input junctions, and 16 outputs.

We calculated the energy consumption of system with varying number of inputs and outputs. The methods for calculating energy consumption and circuit area using integrated circuit design tools are described in the Methods section of the main article.

Supplementary Note 7: comparison with purely CMOS based options

Our approach relies on superparamagnetic tunnel junctions, which convert an analog input into a population of digital spiking outputs, acting as a form of stochastic analog to digital converter. These junctions are associating with digital circuitry and local memory, which implement a learning-capable neural network. Equivalent functionality can be implemented along the same principles entirely with CMOS devices. Different options are summarized and compared in Supplementary Table 1, for the context of a system with 128 inputs and 128 outputs.

The approach closest to our proposal would be to replace the superparamagnetic junctions and their associated read circuitry by analog CMOS spiking neurons that take analog inputs and output digital spikes (Approach 2 in Supplementary Table 1). Many designs for such neurons have been proposed [2]. The most energy efficient versions exploit transistors in the subthreshold regime. As a drawback, such circuits are

prone to device variability, which is compensated by using transistors with high area, even in advanced technology nodes. Using the reference design of [3], the neurons in our design would occupy an area of 1.3 mm², whereas the whole circuit is 0.12 mm² in our approach, and generating the spikes corresponding to one population would consume 330 nJ (0.22 nJ in our approach).

Other entirely CMOS based approaches are possible. All require a conversion between the analog and digital world, which can be performed at various levels of the computations. An approach (Approach 3 in Supplementary Table 1) is to rely on entirely analog non-spiking neurons, such as the ones presented in [4]. Such neurons can be relatively compact and energy efficient. Based on Table I in [4], the neurons would have occupied a more reasonable 1,280 µm² and consumed 200 pJ (assuming the system runs for 10 µs). However, to be processed by digital circuitry, the output of each neuron needs to be converted to a digital output. Extremely compact and energy efficient analog to digital converters (ADCs) have been proposed for low energy contexts, such as [5]. A full conversion requires 20 nJ, and the area of a converter is 0.2 mm². The ADC would therefore be the dominant circuit in terms of area and above all energy consumption, as one conversion per neuron would be required.

A more energy efficient approach would be to use analog non-spiking neurons and perform the computation of the neural network also in the analog domain as suggested in [4] (Approach 4 in Supplementary Table 1). Then, an analog to digital conversion is only required at the output of the computation. The ADC would remain the dominant area and energy consumption in the system, but at only 20 nJ/conversion. A limitation of this approach is that, because it relies entirely on analog computation, it has more limited scalability than approaches using digital neural networks. Also, the memory part of the circuit would be harder to implement[6]. Nevertheless, this remains an attractive option if no access to superparamagnetic tunnel junction is available.

Finally, it is possible to implement entirely digital option, with an ADC directly at the input (Approach 5 in Supplementary Table 1). This approach is highly scalable and requires only one ADC (20 nJ, 0.2 mm²). The computing part can use a similar data path and circuit as the one that we developed for our approach. However, additional digital circuitry is needed to compute a population of neuronal values from the value of the stimulus.

Supplementary Table 1: Comparison with CMOS-only approaches

	Area	Energy	Scalability
**1. Our approach**: Spiking superparamagnetic analog neurons + digital circuitry	Low total area 0.12 mm²	Low energy consumption 0.22 nJ for neurons implementing stochastic ADC plus 6.8 nJ for transformation	Highly scalable
2. CMOS Spiking analog neurons and digital circuitry	High area due to the CMOS spiking neurons > 1.3 mm²	Dominated by input neurons > 330nJ	Highly scalable
3. Non spiking analog neurons and digital circuitry	Significant area due to the ADC after the neurons > 0.2mm²	Dominated by ADC > 2.6µJ (20 nJ/neuron)	Highly scalable

4. Non spiking analog neurons and analog circuitry	Significant area due to the at the output ADC > 0.2mm²	Dominated by ADC > 20nJ	Scalability for medium size circuits
5. Entirely digital circuitry	Significant area due to the ADC at the input > 0.2mm²	Dominated by ADC > 20nJ	Highly scalable

Supplementary Note 8: adaptation of the system to multiple inputs

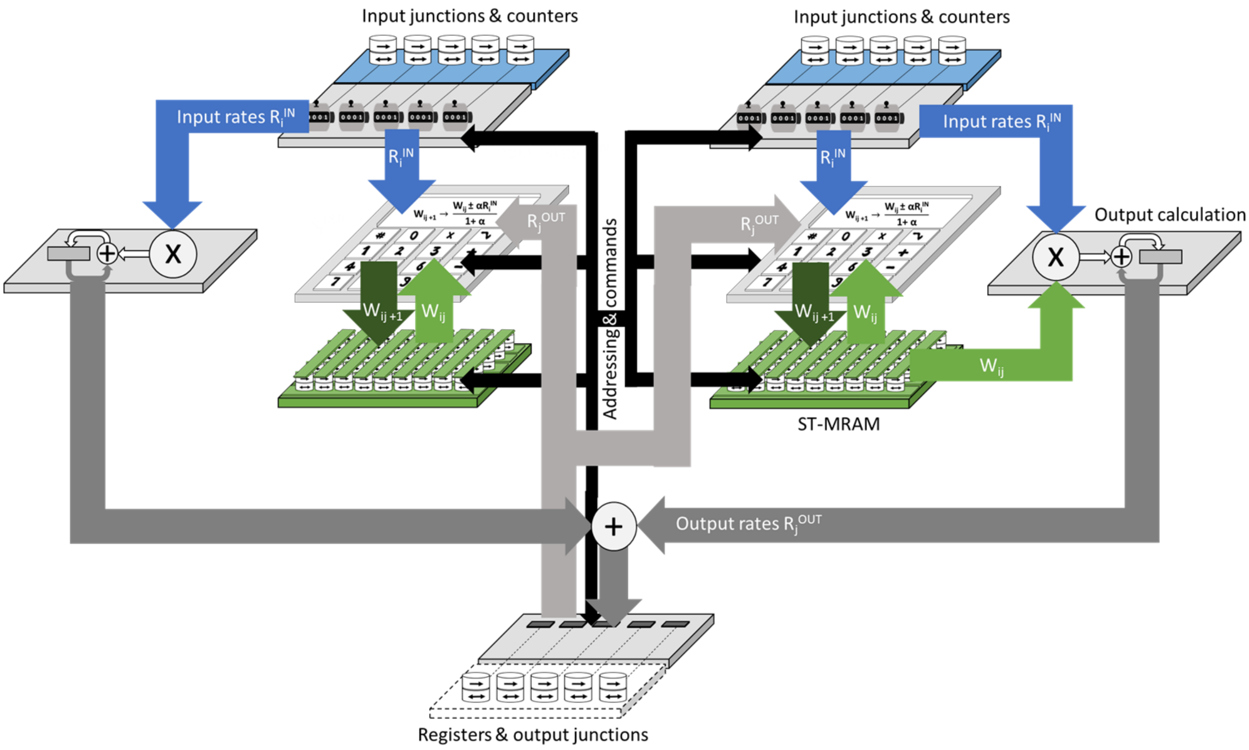

Supplementary Figure 11: Simplified datapath of a system implementing the multi-input architecture presented in Supplementary Fig. 4.

Supplementary References

1. Liu, L., Lee, O. J., Gudmundsen, T. J., Ralph, D. C. & Buhrman, R. A. Current-Induced Switching of Perpendicularly Magnetized Magnetic Layers Using Spin Torque from the Spin Hall Effect. *Phys. Rev. Lett.* **109,** (2012).
2. Indiveri, G. *et al.* Neuromorphic silicon neuron circuits. *Front Neuromorphic Eng.* **5,** 73 (2011).

3. Livi, P. & Indiveri, G. A current-mode conductance-based silicon neuron for address-event neuromorphic systems. in *IEEE Int. Symp. on Circuits and Systems (ISCAS)* 2898–2901 (2009). doi:10.1109/ISCAS.2009.5118408
4. Thakur, C. S., Wang, R., Hamilton, T. J., Tapson, J. & Schaik, A. van. A Low Power Trainable Neuromorphic Integrated Circuit That Is Tolerant to Device Mismatch. *IEEE Trans. Circuits Syst. Regul. Pap.* **63,** 211–221 (2016).
5. Chen, C. H., Zhang, Y., He, T., Chiang, P. Y. & Temes, G. C. A 11 #x03BC;W 250 Hz BW two-step incremental ADC with 100 dB DR and 91 dB SNDR for integrated sensor interfaces. in *Proceedings of the IEEE 2014 Custom Integrated Circuits Conference* 1–4 (2014). doi:10.1109/CICC.2014.6945988
6. Prezioso, M. *et al.* Training and operation of an integrated neuromorphic network based on metal-oxide memristors. *Nature* **521,** 61–64 (2015).
